# Revisiting current “barefoot doctors” in border areas of China: system of services, financial issue and clinical practice prior to introducing integrated management of childhood illness (IMCI)

**DOI:** 10.1186/1471-2458-12-620

**Published:** 2012-08-07

**Authors:** Xiuyun Li, Virasakdi Chongsuvivatwong, Xiaoling Xia, Pasuree Sangsupawanich, Wenjing Zheng, Keling Ma

**Affiliations:** 1 Department of Paediatrics, the Second Affiliated Hospital of Kunming Medical University, Yunnan, PR China; 2 Epidemiology Unit, Faculty of Medicine, Prince of Songkla University, Hat Yai, Thailand; 3 Department of Paediatrics, Faculty of Medicine, Prince of Songkla University, Hat Yai, Thailand

**Keywords:** Village doctor, Integrated management of childhood illness, Health service, Clinical competency

## Abstract

**Background:**

Under-5-years child mortality remains high in rural China. Integrated management of childhood illness (IMCI) was introduced to China in 1998, but only a few rural areas have been included. This study aimed at assessing the current situation of the health system of rural health care and evaluating the clinical competency of village doctors in management of childhood illnesses prior to implementing IMCI programme in remote border rural areas.

**Methods:**

The study was carried out in the border areas of Puer prefecture of Yunnan province. There were 182 village doctors in the list of the health bureau in these border areas. Of these, 154 (84.6%) were recruited into the study. The local health system components were investigated using a qualitative approach and analyzed with triangulation of information from different sources. The clinical component was assessed objectively and quantitatively presented using descriptive statistics.

**Results:**

The study found that the New Rural Cooperative Medical Scheme (NRCMS) coordinated the health insurance system and the provider service through 3 tiers: village doctor, township and county hospitals. The 30 RMB per person per year premium did not cover the referral cost, and thereby decreased the number of referrals. In contrast to available treatment facilities and drug supply, the level of basic medical education of village doctors and township doctors was low. Discontent among village doctors was common, especially concerning low rates of return from the service, exceptions being procedures such as injections, which in fact may create moral hazards to the patients. Direct observation on the assessment and management of paediatric patients by village doctors revealed inadequate history taking and physical examination, inability to detect potentially serious complications, overprescription of injection and antibiotics, and underprescription of oral rehydration salts and poor quality of counseling.

**Conclusion:**

There is a need to improve health finance and clinical competency of the village doctors in the study area.

## Background

During the 1950s, the system of barefoot doctors and the Cooperative Medical Scheme (CMS) financed health care was conducted in China to look after the health of the rural population. This “barefoot doctor” service was initiated by the peasants on a collective and mutual aid basis [1]. After farmers received another medical short-term training (3, 6 or 12 months), they returned to their villages and took on the role of barefoot doctor. Despite simple techniques and inadequate medical equipments at the primary level of health service, the system of barefoot doctor and CMS programme substantially decreased medical costs and provided timely treatment to the rural population [2]. Following the economic reform in the early 1980s, the CMS collapsed and was changed to a fee-for-service system of medical care in rural areas. Coverage of the CMS fell from 90% in the 1960s to 5% in 1985 [3]. At that time, barefoot doctors lost institutional and financial support. The Ministry of Health changed the title of “barefoot doctor” to “village doctor” for those who passed an examination. Those who could not pass turned to other professions [4]. These private village doctors had to focus on treatment of patients to gain financial profit. The level of public health activities in the village decreased. Currently, a farmer who wants to be a village doctor must have at least completed 5 years primary education, and attended and passed the examination of the 1–2 year medical training course at the township or county hospital. Then he/she can be fitted into a vacant position for a village doctor. General education level of village doctor is secondary school and medical health school. Village doctors provide preventive services, maternal and child health services, and simple outpatient care to village residents. Amidst the world trend of health finance reform in 2003, the Chinese government started the New Rural Cooperative Medical Scheme (NRCMS) to reduce the financial burden on rural residents. It is a combined government-run, voluntary, community-based and cost-sharing medical insurance programme. This system requires members of the participating household to pay some part of the premium, including a contribution of 10 RMB, with a local government subsidy of 20 RMB per capita per year [5]. In 2010, NRCMS covered more than 90% of the rural population [6]. However, because of the poor quality of new village doctors who provide primary health care in the village, people are still facing many problems at the grass roots level in China.

Worldwide, almost 10 million children die every year before their fifth birthday [7]*.* Seven in ten of these deaths are caused by acute respiratory infections (mostly pneumonia), diarrhea, measles, malaria, malnutrition or a combination of these conditions [8]. To solve this problem, in the early 1990s the World Health Organization (WHO) and United Nations International Children’s Emergency Fund came up with the Integrated Management of Childhood Illness (IMCI) strategy. The purpose of this programme is to improve the case management in an integrated fashion in order to tackle the most common childhood illnesses [9,10]. Immediately after the introduction of the programme, multi-country evaluations in Uganda, Tanzania, Bangladesh, Brazil and Peru were conducted. It was shown that IMCI could improve the health service quality, and reduce childhood mortality and health care costs. Subsequently, the IMCI programme has been introduced in over 113 countries with varying levels of understanding [10].

Rural China shares the same pattern of childhood diseases listed in IMCI. Pneumonia is still the leading cause of under-five mortality. IMCI was introduced to China in 1998 and since then has been launched as a pilot project in 46 counties of 11 provinces. However, only about 2% of the rural areas have been included [11]. The slow and limited progress of IMCI for more than one decade reflects the low level of commitment by the central and local governments. Over one million village doctors scattered throughout the whole country are still untrained on this important technique.

Yunnan Province is situated in southwest China, bordering Myanmar, Laos and Vietnam and has a population of 45 million. It is one of the poorest and the most remote provinces of China. More than 75% reside in rural areas. In 2001, the provincial average GNP per capita in 2001 was 4,872 RMB (US$609). Approximately 5.6 million people live with an average income per year below the national poverty line of 300 RMB (US$36.1) [12]*.* Living in a mountainous area, rural residents have limited access to health facilities due to the long distances involved in traveling. The high cost of the health care is also a barrier. The mortality rate of children aged under-5-years in border areas (18.6%) was over 5 times higher than that in non-border areas (3.5%) [13].

In 2010, the government of Yunnan planned to launch IMCI training in all rural areas. The highest priority was placed on border areas. This study was conducted prior to the training. It aimed to assess health systems and the clinical competency of village doctors on childhood illness. Information obtained from the study would be used in detailed planning of the training and further improvement of the health system.

## Methods

### Study site

The project was carried out in Puer prefecture, which is the biggest prefecture of Yunnan province. Puer shares borders with Myanmar to the southwest and both Laos and Vietnam to the southeast. There are 9 counties, 103 towns, 994 village committees in the whole prefecture. Among them, there are four counties (Jiangcheng, Ximeng, Lancang and Menglian), 16 townships and 95 village committees in the border areas. The study was conducted immediately before training of village doctors commenced.

### Study design

This was a cross-sectional study combining qualitative and quantitative research. The local health systems and health finance components were investigated using a qualitative approach, whereas the clinical component was assessed objectively and quantitatively. The two components were used to mutually explain each other.

### Study population

The study population was village doctors in border areas in Yunnan province. There were 182 village doctors in the list of the health bureau in these border areas. All of them were invited to undergo training and provide information in this study. Finally, 154 (84.6%) participated the study. Two officers and two doctors in one county hospital and one township hospital, two officers in medical health school were also recruited into the qualitative study part.

### Data collection method and instruments

Data were collected from July to August, 2010. Since the systems of training and management are the same throughout Puer prefecture (as in all other prefectures in China), one county hospital and one township hospital as well as one village clinic in the same chain of supervision were randomly selected and visited to investigate the service systems. The only one local medical health school in Puer where the village doctors had been trained was also visited and the curriculum was reviewed. Emphasis of the review was on how the aforementioned childhood illness classification and management were taught.

For the quantitative part, data were collected by a self-completion questionnaire and a structured observation checklist [14]. The questionnaire consisted of sections on personal demographic characteristics and facilities available at the village clinic. All village doctors were requested to complete it on the day of training registration. Prior to any training, each village doctor’s clinical competency was assessed with one patient exclusively on another day through direct observations by the principal investigator and one assistant researcher, both of whom were experienced paediatricians and IMCI trainers. The checklist was subtly used to record how the village doctors managed the patient. The same patient was re-examined by the principal investigator for gold standard classification and management.

For additional qualitative information, all the recruited village doctors were assembled according to township into 16 groups and further opportunistic individual interviews conducted with the most informative member of each group. To enrich and validate this information, group discussions were additionally carried out with medical health school officers and officers and doctors in county and township hospitals, as mentioned above.

Ethical approval of the study was obtained from the Ethics Committee of the Faculty of Medicine, Prince of Songkla University. Informed verbal consent was obtained from the caregivers of the sick children and all the other participants before data collection.

### Data processing and analysis

R software 2.15 was used for data analysis. Information on health system services and health finance data were analyzed qualitatively with triangulation of information from different sources. Baseline characteristics of village doctors, health facility support system data, clinical competency and patterns of antibiotic prescription were quantitatively measured using descriptive statistics.

## Results and discussion

Village doctors’ characteristics are summarized in Table 1. The majority were male, and were 20–40 years of age. The main ethnicity was Wa. The village doctors had a wide variety of educational background. The two most common levels of education were junior medical college and secondary school alone. Nearly half of all village doctors had worked for more than 10 years.

**Table 1:** Socio-demographic characteristics of village doctors working in border areas of Puer, Yunnan (N = 154)

	**n**	**%**
Gender		
Male	86	55.8
Female	68	44.2
Age (years)		
20-30	53	34.4
31-40	63	40.9
41-50	35	22.7
51-55	3	1.9
Median (IQR)^*^	34.5	(27,40)
Ethnicity		
Wa	69	44.8
Hani	28	18.2
Lahu	21	13.6
Yi	13	8.4
Han	12	7.8
Dai	11	7.1
Lisu	0	0
Bulang	0	0
Education background		
Junior college (3 years after high school)	3	1.9
Medical health school (3 years after secondary school)	54	35.1
Secondary technical school (2 years after secondary school)	7	4.5
High school (11 years)	18	11.7
Secondary school (8 years)	66	42.9
Primary school (5 years)	6	3.9
Employment duration (years)		
Median (IQR) ^*^	9.8	(5, 15)

* IQR: Inter quartile range.

### Health service system

Most of the village doctors were not confident to deal with paediatric patients, especially infants. Common expressions included: “I am afraid to see a small child. When a child patient came to see me, I often referred him/her immediately regardless of the disease”. Also, “I often could not decide whether the child’s disease was severe or not”. A doctor at a township hospital also said: “We cannot deal with children with severe diseases since we are inexperienced. For somewhat severe cases, we will refer them to the county hospital”. Client’s trust is another problem. “A lot of care takers go straight to county hospital because they do not have faith in the competence of village doctors or township hospital doctors”. Self-referral to higher levels often overburdens the care system and is costlier for the caretaker. Consequently, financial barrier is an important gate-keeping mechanism. Since self-referral patients pay the referral fee and get reimbursement at lower rates than a local service, self-referral is limited [15,16].

Under the former CMS, the rural referral system consisted of a three-tier organization for the delivery of health services to its rural populations. Barefoot doctors were attached to the agricultural commune and provided first-level care, whereas second-level care was provided by the township hospital and tertiary care was provided by county and city hospitals [17]. County and township hospitals co-supervised the barefoot doctors. The system was changed by the agricultural reform in the 1980’s but the tiers were ignored. All hospitals became independent and had to ensure their own financial survival. The patients could then go to any level of health services depending on their financial capability. NRCMS has revived the rural 3-tier system. Village doctor, township hospital and county hospital have been re-integrated into a vertical administration to facilitate the referral system. However, more than 60% of the village doctors encountered patients refusing the referral because of their inability to pay. “They often could not go because they could not afford the costs of transportation and relatives’ accommodation and food”. Similar to transportation problems in other developing countries [18], the mountainous terrain of Yunnan makes travelling difficult. “In the rainy season, no vehicle can get out of our village. The road is very boggy, making it difficult for vehicles to go through”. Furthermore, only few township hospitals have an ambulance. If patients in the village need to be referred, they must go by themselves. “There is no referral transportation between village and township hospitals”.

Beliefs in superstition which have been reported in various counties [19] are also a common barrier to rural health care in rural China. “Some severely ill patients think that they have insulted ghosts or spirits, so they just practise certain rituals to gain their pardon instead of going to the hospital”. Referring the patients is therefore often beyond the control of village doctors.

### Human resource quality

The low quality of human resources for rural health has been internationally recognized [20]. Similarly, in China, the most important weakness is at the township hospital level, where patients are referred from the village. Only 5.6% of township hospital doctors in the country are university-trained. Over a half (52.2%) were trained at secondary technical school and medical health school. [21].

Doctors graduating from secondary technical school can attend a distance-learning college refresher course for two or three years to obtain a junior college certificate. Since most have to pay the tuition fee, the participation rate is low and the percentage of absentees from the one month face-to-face classes is very high (estimated at 80%). All who took part in the examination eventually passed and got some promotion on return to work. In this study, 37% of village doctors in Puer and 61% of village doctors in Lincang had formal medical education (including junior college and medical health school) (Table 1). Very few doctors were exposed to paediatric training. Inadequate medical training and lack of paediatric training were mentioned by all doctors. There was about one paediatrician per 100,000 population in the county level. None of them worked at a township hospital.

In reviewing the curriculum of the medical health school of Puer where village doctors had been trained, we found there is no special curriculum for the village doctors. The training materials are all designed for a practitioner working in a city. Major curricula at the medical health school are consistent with those at university with some simplifications. We compared the procedures listed in the textbook used at the medical health school to those covered by IMCI. The items omitted by IMCI include the use of chest X-ray, intravenous injection and more complicated clinical techniques such as using a stethoscope to detect abnormal breath sounds. Ultrasonography, X-ray machines and electrocardiograph are available at most township hospitals. “But no one uses them in our hospital, they are probably not usable”, said a township doctor. For essential drug and equipment/supply in the village clinics, over 80% had specific antimicrobials. However, equally essential supporting items such as Oral Rehydration Salts (ORS), scale and nutritional supplements were available in less than 40% of facilities. Common reasons for not having ORS: “It is not in the list of drugs provided by the township hospital. We are all forbidden to use and store the drug beyond the drug list of NRCMS”. Similarly, growth chart, an important tool for nutritional assessment was not available. No village doctor was aware of its importance.

### Financial mechanism

From the 1950s through to the 1970s, the old CMS was an integrated part of the overall collective system for agriculture production and social services in China. The CMS was primarily financed by the welfare fund of the communes (collective farming). The scheme organized health stations, paid village doctors to deliver primary care, and provided prescription drugs [22]. In the 1980s, around 90% of all peasants were uninsured and inevitably developed catastrophic diseases [23-25]. Despite its current 97% or more coverage, the NRCMS with 30 RMB yearly premiums has a rather “shallow” benefit package. The maximum amount of per annum reimbursement is 200 RMB for each outpatient. The patients pay 55% of these drug costs to the village doctor who then can request the other 45% from the township. If a patient gets a serious disease and is admitted into the township hospital or the county hospital, he/she can get a maximum total reimbursement of 30,000 RMB per year. However, the reimbursement procedure is complicated by bureaucracy [6].

The average salary of the village doctor provided by the local government was 400 RMB per month. This might be generally supplemented if they can complete certain assigned tasks, depending on each county’s regulation. The village doctor purchases drugs from certain drug companies. The costs of the drugs and service fee not included in the list are exclusively under the personal arrangement between the village doctor and the patient. “Our salary is too low. It is not enough even for our transportation”. “When the government set the rule of zero profit for selling drugs, my income diminished. I really don’t want to be a village doctor. If I could find another job, I could earn more money”; “……, we have to get some money from injection fee”. Extra allowance income covers procedures such as injections, acupuncture and massage therapy. In a previous study in Shandong province of China, the percentage of unnecessary items, drug/service and average prescription cost were consistently higher in NRCMS village health stations than in non-NRCMS health stations [26]. This may illustrate the possible role of health insurance on professional moral hazards [27,28]. Regardless of whether moral hazard was the case, in our study, overprescription of drugs and use of unnecessary procedures such as injections could be well explained by lack of clinical competence, elaborated in the following section.

### Clinical competency of village doctors on IMCI checklist

We used the checklist adopted from IMCI to observe the performance of village doctors in this study. The numbers and percentages of doctors who followed the items on the checklist for history taking and physical examination (according to IMCI requirement) are shown in Table 2. Assessment of fever was performed by three quarters of all village doctors, while presence of cough/difficulty breathing was assessed by less than two-thirds and presence of diarrhoea assessed by half. A little over half of the patients were weighed by the village doctors but none of the children’s weight values was checked against the growth chart. Nutritional items were also poorly assessed due to lack of awareness of necessary symptom-specific assessments. Only 17.9% of the village doctors raised the child’s shirt during examination of children with a history of cough/difficulty breathing. Assessment of respiratory rate and chest indrawing were also rarely performed. For children with diarrhoea, 61.1% of village doctors asked for the duration and no village doctor asked about intake of liquids. For patients with fever, 64.3% of village doctors asked for the duration, and none checked for nuchal rigidity (stiff neck), an important sign of meningitis.

**Table 2:** Number and percentage of village doctors’ completeness of history taking and physical examination

	**n**	**%**
**For all children (N = 154)**		
** Completeness of history taking**		
Presence of fever	118	76.6
Presence of cough/difficulty breathing	96	62.3
Presence of diarrhea	79	51.3
History of other problems	33	21.4
Child’s ability to drink or breastfeed	27	17.5
History of severe vomiting	25	16.2
Vaccination status	3	1.9
History of convulsions	0	0
** Physical examination**		
Weight	87	56.5
Presence of palm pallor	5	3.2
Weight checked against the growth chart	0	0
**For children with fever (N = 56)**		
** Completeness of history taking**		
Duration of fever	36	64.3
** Physical examination**		
Temperature	38	67.9
Presence of rash	1	1.8
Nuchal rigidity	0	0
**For children with cough/difficulty breathing (N = 84)**		
** Completeness of history taking**		
Duration of cough	47	64.4
** Physical examination**		
Stridor or wheezing	33	39.3
Raise the child’s shirt when examining	15	17.9
Respiratory rate	6	7.1
Chest indrawing	4	4.8
**For children with diarrhea (N = 36)**		
** Completeness of history taking**		
Ask for duration	22	61.1
Ask for blood in stool	18	50.0
** Physical examination**		
Look for sunken eyes or fonticulus	3	8.3
Pinch the skin of the abdomen	0	0
Intake of liquids	0	0

Table 3 compares the clinical classifications and management of the patients as assessed by the village doctors with that of the certified paediatrician (assumed to be the gold standard). The paediatrician noted 231 classifications among the 154 patients, half of them (50.2%) missed or incorrectly classified by the village doctors, including 2 cases of pneumonia, 2 cases of severe pneumonia and 3 cases of diarrhea with dehydration. Thus around 5% of all patients, or around two-thirds of the severe patients, would have progressed to more serious conditions had they not been reassessed by the paediatrician.

**Table 3:** Frequency of disease classification, management and counseling given by village doctors compared with gold standard

	**Gold standard**	**Village doctor**
Classification*		
Total	231	115
Cough, without pneumonia	98	71
Pneumonia	4	2
Severe pneumonia	2	0
Diarrhea without dehydration	31	1
Diarrhea with dehydration	5	2
Fever	56	22
Ear problems	1	1
Malnutrition	7	4
Others	27	12
**Treatment considered as necessary**		
Antibiotics	21	112
Injectable drugs	7	87
ORS	27	2
Referral	5	2
**Counseling to caretakers**		
How to take medicine	154	52
Give extra fluids	154	15
When to return immediately	154	9
Verification of comprehension	154	15

* Some patients with more than one classification.

According to the paediatrician’s assessment, only 13.6% of the patients needed antibiotics compared to 72.7% as determined by village doctors. Only 4.5% needed injection drug, but 56.5% of patients were deemed to need an injection by village doctors, and among these, most (97.7%) were for antibiotics. While antibiotics were overprescribed in this study, the level of supportive care was found to be inadequate. Of the 27 patients requiring ORS, only 2 were prescribed with it. This was despite the emphasis of ORS use in the curriculum in the medical health school. The finding is of considerable concern. There might be two reasons explaining the misuse of ORS. One is that ORS was not included in the drug list under NRCMS, and government forbids village doctors to sell medicine outside the drug list. Another is that few village doctors knew the guideline to treat diarrhea and how to use ORS. Counseling for patient home care, which was necessary in all patients, was given by only one third of village doctors. How to give oral medicine was the most common (33.8%) counseling given. Advice on giving extra fluids, recognizing the signs that necessitate immediate return to the village doctor and verification of the care taker’s comprehension of these advices were given to less than 10% of patients.

Antibiotics prescribed by village doctors are summarized in Table 4. The most commonly prescribed injectable and oral antibiotics were penicillin and amoxicillin, respectively. Third generation cephalosporins, suitable for highly resistant hospital acquired infections, were prescribed in 8.4% of patients by either or both routes. Combined antibiotics with antiviral were prescribed in 16.8% of patients. Overprescription of antibiotics and injectable drugs found in this study has been similarly reported by many other investigators [29-35]. Provider’s perception and knowledge about antibiotic use impacts the pattern of prescription [36]. Studies showed that IMCI training can reduce the inappropriate prescription for childhood illness [11,37]. However, education alone will not be enough, a view confirmed by the experience of applying Australian guidelines in Chinese hospitals [38]. Health providers faced financial incentives to prescribe, with profit splitting with pharmaceutical suppliers [36]. A review study also mentioned that China’s fee-for-service payment system has resulted in poor quality of services and overprescriptions by village doctors [39]. Our study also found the inappropriate incentives of village doctors’ overprescriptions for childhood illness. While antibiotics are important to treat childhood infections, overuse may cause antibiotic resistance among common bacteria in China [40,41]. Common serious infections, such as pneumonia resulting from antibiotic resistant organisms, are considered to be reemerging infectious diseases of global importance [42]. Attempts to control antibiotic overuse cannot be overemphasized. 

**Table 4:** Summary of antibiotics prescribed by village doctors during the competency assessment (N = 154)

	**n**	**%**
Injectable antibiotics*	**85**	**55.2**
Penicillin	39	25.3
First generation cephalosporin	31	20.1
Third generation cephalosporin	11	7.1
Metronidazole	9	5.8
Others	5	3.2
**Pattern of use**		
Only one antibiotic	75	48.7
More than one antibiotic	10	6.5
**Combination with other medicine**		
Alone	57	37.0
With antiviral	16	10.4
With traditional Chinese medicine (TCM**)	10	6.5
With antiviral and TCM**	2	1.3
Oral antibiotics*	**79**	**51.3**
Amoxicillin	69	44.8
First generation cephalosporin	4	2.6
Roxithromycin	3	1.9
Gentamicin	3	1.9
Third generation cephalosporin	2	1.3
Metronidazole	2	1.3
**Pattern of use**		
Only one antibiotic	70	45.5
More than one antibiotic	9	5.8
**Combination with other medicine**		
Alone	34	22.1
With TCM**	37	24.0
With antiviral	6	3.9
With antiviral and TCM**	2	1.3
**Injectable + oral antibiotics**	**52**	**33.8**

* Responses are not mutually exclusive.

** Traditional Chinese Medicine.

Without prior IMCI training, it is not surprising that most aspects of case management were incorrect. Past training in the regular curriculum did not provide the village doctor with essential clinical competency. Lack of ability to detect serious childhood conditions among these primary health care workers may partially explain the high mortality rate of children in remote rural China. It will be interesting to see the effectiveness of the IMCI training program in reducing the clinical incompetence and over prescription of antibiotics in childhood illness among village doctors.

### Limitation of these findings

Our data were confined to only one prefecture of Yunnan. Generalization of other areas must be interpreted with caution.

## Conclusions

Control of common childhood illnesses in rural China is in the hands of the rural health care system and current village doctors who evolved from “barefoot doctors” and the 3-tier system sixty years ago. This limited study revealed that at least in the study border area, the rural health care system now has modern equipment and a relatively adequate drug supply. The major barriers are in people’s financial situation, motivation of the village doctors and their clinical competence. Both health finance reform and improvement of clinical competency of the village doctors must be accelerated.

## Abbreviations

CMS: Cooperative Medical Scheme; IMCI: Integrated management of childhood illness; NRCMS: New Rural Cooperative Medical Scheme; ORS: Oral Rehydration Salts; WHO: World Health Organization.

## Competing interests

The authors declare that they have no competing interests.

## Authors’ contributions

XL was principal investigator of the study, conceptualized the research, collected the data, performed data analysis, and drafted the manuscript. VC conceived the study, assisted in development on manuscript writing, and provided supervision and suggestions. XX participated in its design, coordination and helped to draft the manuscript. PS provided supervision and suggestions and helped to draft the manuscript. WZ helped to draft the manuscript. KM helped to draft the manuscript. All authors read and approved the final manuscript.

## Pre-publication history

The pre-publication history for this paper can be accessed here:

http://www.biomedcentral.com/1471-2458/12/620/prepub
